# Socioeconomic disparity trends in diagnostic imaging, treatments, and survival for non‐small cell lung cancer 2007‐2016

**DOI:** 10.1002/cam4.2978

**Published:** 2020-03-20

**Authors:** Monica Shah, Ambica Parmar, Kelvin K. W. Chan

**Affiliations:** ^1^ Faculty of Medicine University of Toronto Toronto ON Canada; ^2^ Odette Cancer Centre Sunnybrook Health Sciences Centre Toronto ON Canada; ^3^ Canadian Centre for Applied Research in Cancer Control Toronto ON Canada; ^4^ Cancer Care Ontario Toronto ON Canada

**Keywords:** cancer stage, cancer treatment, diagnostic test, imaging, lung cancer, overall survival, socioeconomic status, trend

## Abstract

Socioeconomic status (SES) has led to treatment and survival disparities; however, limited data exist for non‐small cell lung cancer (NSCLC). This study investigates the impact of SES on NSCLC diagnostic imaging, treatment, and overall survival (OS), and describes temporal disparity trends. The Ontario Cancer Registry was used to identify NSCLC patients diagnosed between 2007 and 2016. Through linkage to administrative datasets, patients’ demographics, imaging, treatment, and survival were obtained. Based on median household neighborhood income, the Ontario population was divided into five income quintiles (Q1‐Q5; Q1 = lowest income). Multivariable regressions assessed SES association with OS, imaging, treatment receipt, and treatment delay, and their interaction with year of diagnosis to understand temporal trends. Endpoints were adjusted for demographics, stage and comorbidities, along with treatments and imaging for OS. A total of 50 542 patients were identified. Higher SES patients (Q5 vs. Q1) showed improved 5‐year OS (hazard ratio, 0.89; 95% confidence interval [CI], 0.87‐0.92; *P* < .0001) and underwent greater magnetic resonance imaging head (stages IA‐IV; odds ratio [OR], 1.24; 95% CI, 1.16‐1.32; *P* < .0001), lung resection (IA‐IIIA; OR, 1.58; 95% CI, 1.43‐1.74; *P* < .0001), platinum‐based vinorelbine adjuvant chemotherapy (IB‐IIIA; OR, 1.63; 95% CI, 1.39‐1.92; *P* < .0001), palliative radiation (IV; OR, 1.14; 95% CI, 1.05‐1.25; *P* = .023), and intravenous chemotherapy (IV; OR, 1.45; 95% CI, 1.32‐1.60; *P* < .0001). Lower SES patients underwent greater thoracic radiation (IA‐IIIB; OR, 0.86; 95% CI, 0.79‐0.94; *P* = .0003). Across 2007‐2016, socioeconomic disparities remain largely unchanged (interaction *P* > .05) despite widening income inequality.

## Graphical abstract

Longstanding socioeconomic disparities in non‐small cell lung cancer imaging, treatments, and survival persist without improvement despite a universal health care system. Although these socioeconomic disparities remain largely unchanged over time, further exploratory research is necessary to better understand their causal pathways in efforts to reduce these inequalities.

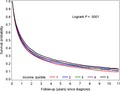

## 1 INTRODUCTION

Lung cancer accounts for 25.4% of all cancer cases worldwide.^1^ In Canada, it is the second most common cancer type and the greatest cause of cancer‐related death, with non‐small cell lung cancer: (NSCLC) constituting the majority.^2^

Lower socioeconomic status (SES) is known to be associated with increased incidence^3^, ^4^ and poorer lung cancer survival,^5^, ^6^, ^7^, ^8^, ^9^, ^10^, ^11^ even within countries providing universal health care coverage, such as Canada.^3^, ^4^, ^5^, ^8^ However, limited research has controlled for cancer‐specific factors including imaging and treatment when exploring NSCLC overall survival (OS).

The precise use of imaging is integral to ensure adequate staging and the appropriate delivery of advanced curative intent treatment for NSCLC.^12^, ^13^ Socioeconomic status may negatively influence the receipt of adequate imaging.^14^, ^15^, ^16^ Okafor et al have shown higher SES as a predictor for computed tomography (CT) and endoscopic ultrasound receipt in rectal cancer, with the latter associated with higher use of neoadjuvant chemoradiation.^14^ To date, no studies have examined the frequency of diagnostic imaging based on SES in NSCLC.

Low SES has also led to delays in the time from diagnosis to first treatment in various cancer types,^17^, ^18^, ^19^ known as time‐to‐treatment initiation (TTI).^20^ Additionally, the receipt of appropriate treatments is less likely in NSCLC patients with lower SES.^6^, ^21^, ^22^ While literature has shown treatment to be associated with SES, conflicting findings exist for NSCLC treatment delay^23^, ^24^ and few studies have examined the association between SES and NSCLC patterns of care *over time*. Current research has solely explored temporal trends in the association between SES and survival.^25^, ^26^ Dabbikeh et al have demonstrated less improvement in lung cancer‐specific survival among patients from poorer communities in Ontario between 1993 and 2009.^26^

In Canada, the Gini coefficient, which measures income inequality (a value of 0 expressing perfect equality and 1 expressing total inequality), moved from a low of 0.28 in 1989 to 0.32 in 2010.^27^ As income inequality has been increasing in many countries such as Canada, it is important to understand whether these changes are translating into further disparities in the diagnostic work‐up, treatment and outcomes associated with NSCLC. As well, it is important to examine OS independent of patient and cancer‐specific factors including imaging and treatment, that greatly impact survival and vary across SES groups.^5^, ^28^, ^29^

The objectives of the current study were thereby to examine the impact of SES on diagnostic imaging, treatment receipt, TTI, time to adjuvant treatment, and OS rates in Ontario patients with NSCLC, as well as to examine temporal changes in these socioeconomic disparities across 2007‐2016.

## 2 MATERIALS AND METHODS

### 2.1 Design and data collection

This was a population‐based retrospective cohort study. Through the Registered Persons Database (RPDB) and the Ontario Cancer Registry (OCR), we identified all NSCLC cancer cases in Ontario between 1 January 2007 and 31 December 2016, and their demographics and diagnostic information including age, sex, postal code at diagnosis, date of diagnosis, disease site, and date of death. All adult patients (age > 18 years) with TNM staging at diagnosis available were included. Classification of malignant tumors based on the American Joint Committee on Cancer (AJCC) TNM staging 7th edition was used in the OCR from 2007 onwards. Cases not confirmed microscopically, benign neoplasms and small cell lung cancer types were excluded. Histopathological NSCLC cancer diagnosis was determined by the International Classification of Diseases (ICD) morphology codes (10th edition).

Information regarding the use of diagnostic imaging [CT, positron emission tomography (PET), bone scan, magnetic resonance imaging (MRI)], receipt of treatment therapies (surgery, chemotherapy, radiotherapy), TTI (defined as the time from diagnosis to first treatment receipt), and time to adjuvant treatment for NSCLC patients was gathered from the following five sources: the Ontario Health Insurance Plan, the Canadian Institute for Health Information Discharge Abstract Database, the New Drug Funding Program, the National Ambulatory Care Reporting System, and the Ontario Drug Benefit database (Table S1). All the above treatment and imaging services are publicly funded and administered in Ontario.

### 2.2 Outcome variables

Outcome measures involved within the analysis include cancer stage, OS, and patterns of care. OS is defined as the time between NSCLC diagnosis and time of patient death from any cause until 31 December 2016. Patterns of care outcome variables include frequency of NSCLC imaging (CT head, MRI head, bone scan, and PET for all stages), treatment receipt [lung resection surgery for stages IA‐IIIA, platinum‐based vinorelbine adjuvant chemotherapy for stages IB‐IIIA, thoracic radiation for stages IA‐IIIB, palliative radiation for stage IV, intravenous (IV) chemotherapy for stage IV, and epidermal growth factor receptor inhibitor (EGFR‐I) for stage IV], TTI (time between diagnosis and lung resection surgery for stages IA‐IIIA, and time between diagnosis and thoracic radiation for stages IA‐IIIB), and time to adjuvant treatment (time between surgery and platinum‐based vinorelbine adjuvant chemotherapy for stages IB‐IIIA).

As MRI brain performance and surgical resection are less commonly performed in stages IA and IIIA, respectively, to examine the robustness of our results, additional sensitivity analyses were conducted for receipt of MRI head restricted to stages IB‐IV, receipt of lung resection surgery restricted to stages IA‐IIB, and time between diagnosis and lung resection surgery restricted to stages IA‐IIB.

Bone scan, MRI head, CT head, and PET were examined within 90 days peri‐diagnosis, lung resection surgery and thoracic radiation within 180 days postdiagnosis, adjuvant vinorelbine within 180 days postsurgery, and EGFR‐I, palliative radiation, and IV chemotherapy at any time postdiagnosis.

### 2.3 Predictor variables

Median neighborhood household income, indicated by patients’ postal codes, was used to determine area‐level SES.^26^, ^30^ Patients’ postal codes were linked to data from the Canadian censuses. Income was categorized into five quintiles corresponding to neighborhood income status. Income quintile five represents the wealthiest 20% of neighborhoods, and income quintile one represents where the poorest 20% reside.

For both the diagnostic imaging and treatment receipt models, patient‐level predictor variables that were adjusted for include: age, sex, cancer stage, rural versus urban geographic location, and comorbidities via Aggregated Disease Groups (ADG) and Charlson Comorbidity Index, including prior chronic obstructive pulmonary disease (COPD), hypertension, prior myocardial infarction (MI), prior congestive heart failure (CHF), and diabetes.

For the OS model, both the aforementioned patient‐level predictor variables, as well as diagnostic imaging and treatment receipt were adjusted for.

### 2.4 Statistical analyses

Summary statistics (mean, median, proportions) and tests including chi‐square, Cochran‐Armitage trend for categorical variables, and Jonckheere‐Terpstra trend for continuous variables were used to evaluate and compare patient demographics across SES groups.

Both univariable and multivariable logistic and linear regression models were used to determine associations between SES with: (a) cancer stage at diagnosis, (b) MRI head use, (c) PET use, (d) bone scan use, (e) CT head use, (f) receipt of lung resection surgery, (g) receipt of thoracic radiation, (h) receipt of adjuvant vinorelbine, (i) receipt of IV chemotherapy for stage IV disease, (j) receipt of EGFR‐I for stage IV disease, (k) time between diagnosis and lung resection surgery, (l) time between diagnosis and thoracic radiation, and (m) time between surgery and adjuvant vinorelbine.

Overall survival was calculated from initial date of diagnosis to date of death from all causes. The Kaplan‐Meier (KM) method was undertaken to aid in examining the relation between SES and OS. Univariable and multivariable proportional hazards regressions were also used to investigate the effect between SES and OS.

To assess temporal trends of SES and outcomes in year of diagnosis between 2007 and 2016, *P*‐values for interaction terms between income quintile and index year were examined, treating income quintile and year of diagnosis as continuous variables.

Results were considered significant if *P *< .05, and all tests of statistical significance were two‐sided. Statistical analysis was performed using SAS version 9.4 (SAS Institute Inc).

### 2.5 Ethics approval

Study approval was granted by the Research Ethics Board at Sunnybrook Research Institute.

## 3 RESULTS

### 3.1 Patient demographics

**FIGURE 1:**
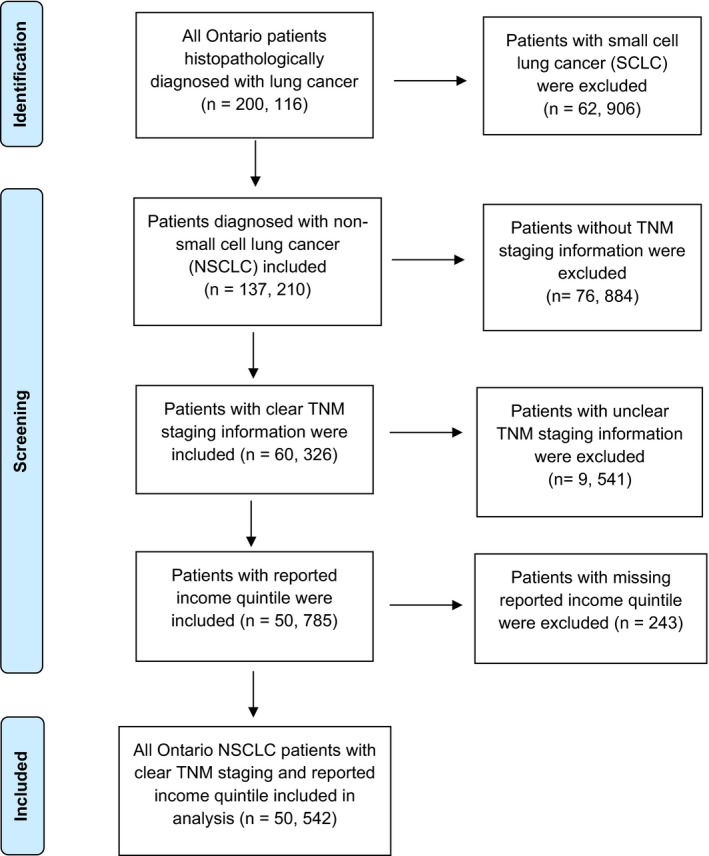
Flow diagram of included patients. NSCLC: non‐small cell lung cancer; SCLC: small cell lung cancer; TNM: Tumor, node, metastasis

We identified a total of 50, 542 patients (48.1% female, 51.9% male) diagnosed with NSCLC (Figure 1). In total, 68% of patients were over the age of 65, 15.2% of patients lived in a rural environment, and 21.1%, 53.3% and 25.6% had ADG scores between 0‐4, 5‐9, and ≥10, respectively. Table 1 illustrates patients' demographics, comorbidities and cancer stage at diagnosis, expressed as a percentage of patients in each income quintile.

**TABLE 1:** Patient demographics, comorbidities, and initial cancer stage at diagnosis, expressed as a percentage of patients in each income quintile

Variable	Percentage of patients						*P*
	Total	Q1 (Lowest), n = 11 609 (23.0%)	Q2, n = 11 015 21.8%)	Q3, n = 9915 19.6%)	Q4, n = 9625 19.0%)	Q5, n = 8378 (16.6%)	
Age ≥ 65	68.0	66.3	67.8	67.9	68.4	70.0	<.0001***
Sex (Male)	51.9	51.3	52.0	52.5	53.1	50.8	.011*
Comorbidities (ADG)							<.0001***
0‐4	21.1	22.4	21.5	20.8	20.5	19.9	
5‐9	53.3	51.3	53.3	54.7	54.2	53.5	
≥10	25.6	26.4	25.3	24.5	25.3	26.6	
Rural	15.2	16.2	14.8	15.4	14.8	14.7	.012*
Stage							.003**
IA	13.1	12.8	12.7	13.7	12.7	14.0	
IB	9.7	9.3	9.5	9.9	10.2	9.7	
IIA	3.8	3.8	3.8	3.6	3.8	4.2	
IIB	4.7	5.1	4.9	4.3	4.6	4.3	
IIIA	12.7	13.2	13.0	12.1	12.4	12.5	
IIIB	9.1	9.4	9.1	9.1	9.4	8.4	
IV	46.9	46.3	47.1	47.5	46.8	46.8	

Note

Q indicates income quintile.

*Denotes a statistically significant finding (*P < .05. **P < .01. ***P < .001) where *P * is from chi‐squared.

### 3.2 Patterns of care receipt

**FIGURE 2:**
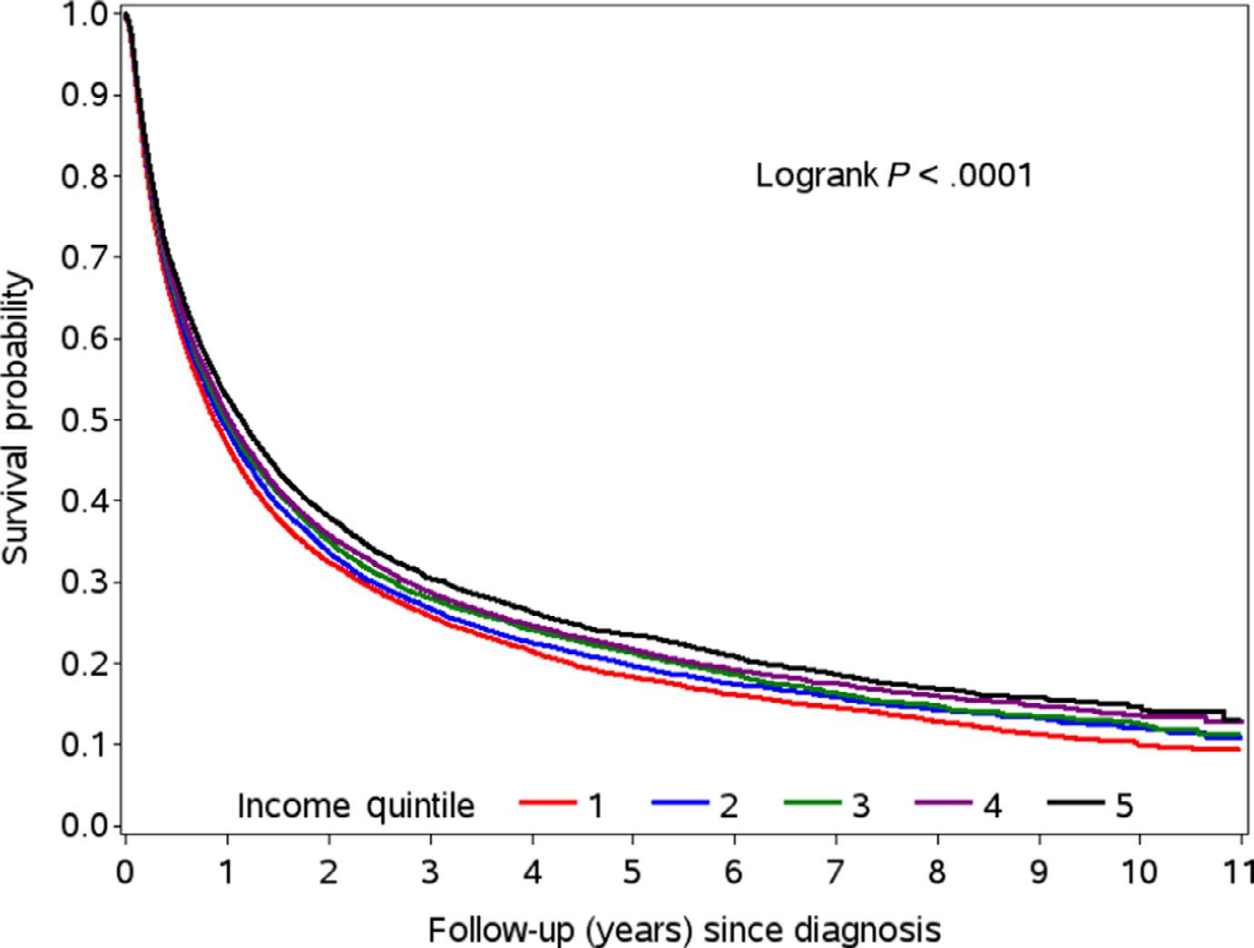
Overall survival curves for non‐small cell lung cancer patients within each SES quintile (Q1‐Q5)

Table 2 summarizes diagnostic imaging and treatment receipt by SES quintile. Table 3 outlines the average times (in days) from diagnosis to first treatment, and to adjuvant treatment. With multivariable analyses we found that patients with higher SES were more likely to undergo MRI head (stages IA‐IV; Q5 vs. Q1: odds ratio [OR], 1.24; 95% confidence interval [CI], 1.16‐1.32; *P *< .0001), lung resection surgery (stages IA‐IIIA; Q5 vs. Q1: OR, 1.58; 95% CI, 1.43‐1.74; *P *< .0001), adjuvant vinorelbine (stages IB‐IIIA; Q5 vs. Q1: OR, 1.63; 95% CI, 1.39‐1.92; *P *< .0001), palliative radiation (stage IV; Q5 vs. Q1: OR, 1.14; 95% CI, 1.05‐1.25; *P* = .023) and IV chemotherapy (stage IV; Q5 vs. Q1: OR, 1.45; 95% CI, 1.32‐1.60; *P *< .0001). Higher SES patients also showed better 5‐year OS (Q5 vs. Q1: hazard ratio, 0.89; 95% CI, 0.87‐0.92; *P *< .0001). The adjusted 5‐year OS KM estimates for income quintiles Q1‐Q5 were 20.0%, 20.8%, 21.0%, 21.2%, and 21.8%, respectively (see Figure 2). In contrast, lower SES patients underwent greater thoracic radiation (stages IA‐IIIB; Q5 vs. Q1: OR, 0.86; 95% CI, 0.79‐0.94; *P *= .0003).

**TABLE 2:** Patterns of care for the patient population with available data on stages I‐IV non‐small cell lung cancer, expressed as a percentage of patients in each income quintile

Patterns of care variable	Percentage of patients					OR^a^	*P* ^b^	*P* for interaction (with time)^c^
	Q1	Q2	Q3	Q4	Q5			
All stages								
CT head	46.1	46.4	46.7	46.6	45.9	0.99	.88	.041*
MRI head	29.6	30.4	31.0	32.1	34.0	1.24	<.0001***	.69
Bone scan	51.2	52.7	52.5	51.8	50.5	0.99	.13	.012*
PET	28.9	28.8	29.3	29.9	30.4	1.01	.054	.50
Stages IB‐IV								
MRI head (sensitivity analysis)	30.0	30.8	31.3	32.6	34.4	1.25	<.0001***	.69
Stages IA‐IIIA								
Lung resection surgery	46.8	51.1	53.6	56.1	57.0	1.58	<.0001***	.34
Stages IA‐IIB								
Lung resection surgery (sensitivity analysis)	58.1	62.7	64.3	68.3	68.2	1.56	<.0001***	.19
Stages IA‐IIIB								
Thoracic radiation	34.9	33.1	32.2	30.6	30.5	0.86	.0003***	.16
Stages IB‐IIIA								
Platinum‐based vinorelbine adjuvant chemotherapy	11.4	12.0	13.9	14.5	15.8	1.63	<.0001***	.19
Stage IV								
Palliative radiation	63.7	65.2	65.8	66.0	66.2	1.14	.023*	.95
IV chemotherapy	26.2	29.2	31.0	32.2	33.0	1.45	<.0001***	.70
EGFR inhibitor	7.3	7.7	7.8	8.8	9.3	1.20	.064	.40

Note

Q indicates income quintile.

Abbreviations: CT, computed tomography; EGFR, epidermal growth factor receptor; IV, intravenous; MRI, magnetic resonance imaging; PET, positron emission tomography; SES, socioeconomic status.

^a^ adjusted OR with Q5 versus Q1.

^b^ *P*‐value for the multivariable regression model effect of SES and outcome, without the interaction term between SES and time.

^c^ *P*‐value for the interaction term between income quintile and index year, in the multivariable regression model.

* Denotes a statistically significant finding (*P < .05. ***P < .001) where *P* is the overall income quintile adjusted wald chi‐squared test from the logistic regression model.

**TABLE 3:** Mean time‐to‐treatment initiation and time to adjuvant treatment for the patient population with Stages IA‐IIIB non‐small cell lung cancer, expressed in days

Patterns of care variable	Mean time (days)					*P* ^a^	*P* for interaction (with time)^b^
	Q1	Q2	Q3	Q4	Q5		
Stages IA‐IIIA							
Lung resection surgery following diagnosis	51.5	50.1	49.5	48.2	41.2	.10	.40
Stages IA‐IIB							
Lung resection surgery following diagnosis (sensitivity analysis)	49.1	48.7	46.8	45.8	46.5	.04*	1.00
Stages IA‐IIIB							
Thoracic radiation following diagnosis	74.1	72.7	74.1	72.2	70.7	.16	.98
Stages IB‐IIIA							
Platinum‐based vinorelbine adjuvant chemotherapy following surgery	62.1	64.2	63.3	62.7	61.6	.41	.42

Note

Q indicates income quintile.

^a^ *P*‐value for the multivariable regression model effect of SES and outcome, without the interaction term between socioeconomic status (SES) and time.

^b^ *P*‐value for the interaction term between income quintile and index year, in the multivariable regression model.

* Denotes a statistically significant finding (*P* < .05) where *P* is the overall adjusted income quintile F value from multivariable linear regression.

Statistical significance remained unchanged for the receipt of MRI head for stages IA‐IV (*P* < .0001) in comparison to stages IB‐IV in the sensitivity analysis (*P* < .0001), as well as for the receipt of lung resection surgery for stages IA‐IIIA (*P* < .0001) in comparison to stages IA‐IIB in the sensitivity analysis (*P* < .0001). New statistical significance, however, was found for mean time to lung resection surgery following diagnosis for stages IA‐IIB (*P* = .04) opposed to stages IA‐IIIA (*P* = .10).

### 3.3 Temporal trends

Across 2007‐2016, there was a borderline decreased effect of SES quintile on bone scan receipt (interaction *P* = .012) and a borderline increased effect of SES quintile on CT head receipt (interaction *P* = .041). There were no substantial changes in the receipt of other diagnostic imaging techniques (MRI head, PET), nor treatments (lung resection surgery, thoracic radiation, palliative radiation, IV chemotherapy, adjuvant vinorelbine, EGFR‐I) over time based on SES. Examining both TTI (lung resection surgery following diagnosis, thoracic radiation following diagnosis) and time to adjuvant treatment (adjuvant vinorelbine following surgery), there were no significant effects of SES found across 2007‐2016, regardless of the year of diagnosis. While lower SES groups demonstrated lower OS rates, over time, there was also no increase nor decrease in this socioeconomic disparity (interaction *P* = .62). Temporal trends for diagnostic work‐up, treatment receipt, and outcomes are reviewed in Tables 2 and 3.

## 4 DISCUSSION

Irrespective of Ontario's mostly publicly funded health care system, this study demonstrated that higher SES is associated with greater diagnostic imaging, treatment receipt and OS rates in NSCLC patients. Fortunately, socioeconomic disparities across survey years remain generally unchanged despite widening income inequality.

Examining the distribution of patients across income quintiles, with 23.0% of all NSCLC patients falling into income Q1% versus 16.6% in Q5, it is evident that patients with lower SES have higher rates of lung cancer diagnosis. This is consistent with previous findings.^31^, ^32^ Wong et al report an inverse relationship between NSCLC incidence and SES for white women, as well as white, black, Asian, and Pacific Islander men.^31^

Higher SES NSCLC patients underwent greater lung resection surgery in stages IA‐IIIA, adjuvant vinorelbine in stages IB‐IIIA, and IV chemotherapy and palliative radiation in stage IV. These findings are in line with previous literature showcasing an effect of income on treatments.^6^, ^21^, ^22^ Yorio et al demonstrated that socioeconomically disadvantaged patients with stages I‐III NSCLC were less likely to undergo standard surgery and/or radiation therapy.^21^ Furthermore, higher SES patients were more likely to undergo MRI head in stages IA‐IV, similar to findings by Leapman et al showcasing that highest SES patients were more likely to receive diagnostic MRI for prostate cancer.^16^ These findings support a need to increase diagnostic imaging and treatment rates when appropriately indicated among the impoverished, especially as predictors of OS rates. Similar to previous literature,^25^, ^29^ higher SES patients were found to have increased OS rates (Q1 = 20.0% vs. Q5 = 21.8%) after adjusting for confounders, although differences are small and may not be clinically meaningful despite achieving statistical significance.

Dissimilar to diagnostic imaging and treatment receipt, no association was found between SES and TTI (aside from the sensitivity analysis), nor time to adjuvant treatment. Within the sensitivity analysis our findings suggest that higher SES patients experienced shorter time delays between diagnosis and receipt of surgery (*P* = .04). Despite statistical significance, however, the spread of time in days varied little (a maximum difference of 5.3 days between Q1 and Q5); as such, the result is not clinically meaningful. We hypothesize that the reason time results do not differ across income quintiles while treatment receipt does, is because the analysis of TTI and time to adjuvant treatment itself has already selected for individuals where treatment receipt is not an issue. Although similar findings by Moriceau et al show no significant difference in wait times between diagnosis and treatment for lung cancer,^24^ contrasting findings by Nadpara et al demonstrate that lower SES groups experienced greater delays in TTI.^23^ With conflicting findings and limited research on NSLSC treatment delay, it is important for further research to be conducted.

Furthermore, across 2007‐2016 the disparity between highest and lowest SES quintiles slightly decreased across increasing year of diagnosis for bone scan receipt (interaction *P* = .012), and slightly increased across increasing year of diagnosis for CT head (interaction *P* = .041). However, the interaction parameter estimates for both bone scan and CT are only 0.05% of that of their respective income quintile parameter estimates, demonstrating trivial clinical significance. Moreover, there were no substantial changes in the receipt of MRI head, PET, lung resection surgery, thoracic or palliative radiation, IV chemotherapy, adjuvant vinorelbine, nor EGFR‐I, along with OS rates, TTI and time to adjuvant treatment over time based on SES. While it is optimistic that socioeconomic gaps are not worsening over time despite widening income inequality, their persistence in today's modern era may be concerning. Many factors may help to explain why socioeconomic disparities are not diminishing. Lower SES individuals have been found to experience more frequent physical work demands, monotony, low autonomy, and inflexibility of work hours, leading to financial stress and job insecurity.^33^ As low SES individuals may work several jobs to meet financial demands, it is important to note that longer working hours and opportunity costs associated with attending medical appointments, may act as nonfinancial barriers to accessing care.^34^, ^35^, ^36^ This is especially important as longer working hours can lead to poorer health outcomes in low SES groups.^37^ Conway et al showed that individuals working 52+ hours per week for a minimum of 10 years, in comparison to 35‐51 hours, had higher risks of cancer.^38^ If low SES individuals are worried about their financial situations and are unable to take more time off work, they will not be able to access valuable health resources even within a universal health care system.

Additional factors may also contribute to the noted disparities in cancer care. Previous studies have found low SES to be more common among non‐white races,^39^ and have found evidence of racial disparities in NSCLC treatment, with Hispanics and Blacks receiving fewer operations and less timely care.^20^, ^40^ Moreover, higher education levels are associated with improved outcomes for NSCLC,^41^ and increased awareness regarding the availability and benefits of imaging and treatments.^30^, ^42^ Although race and education level may contribute to the lower use of MRI and treatments within the lowest SES quintile, efforts to stratify patients based on these variables were not possible due to the limitations of Ontario population‐based databases.

Furthermore, CT head and bone scan are becoming less commonly used with increasing widespread use of PET‐CT and MRI head. Nevertheless, given the timeline for our study examining years 2007‐2016 (over a decade), we examined the totality of evidence by including CT head and bone scan in addition to MRI and PET‐CT.

Another limitation may be the operative definition of SES. Previous studies have validated using average neighborhood income through patient postal code as an inexpensive and readily available method to calculate patient income for all persons regardless of age, sex, or employment status.^43^, ^44^ Although this method may reduce response bias from incomplete or inaccurate individual‐level socioeconomic information, it may be less sensitive than individual measures of income for each patient.^43^, ^44^ Inherent to the study of SES, randomized design, and thereby matched baseline characteristics, were also infeasible. Subsequently, patients may have had competing risks of mortality from other diagnoses aside from NSCLC, such that we cannot fully attribute OS to lung cancer‐related events.

Furthermore, we were unable to obtain smoking status, which has been found to be associated with poorer survival in early stage NSCLC.^45^ However, COPD was controlled for, for which smoking is a large predicting factor and produces a synergistic effect that leads to four times greater risk of lung cancer.^46^

Last, as this study was a population‐based analysis of Ontario, it may not be generalizable outside of Canada given differences in both health care access and health care funding model (eg, public funding). The differences reported across socioeconomic groups may be potentially worse in jurisdictions without universal health care. However, as Ontario is the most populous province within Canada representing 35%‐40% of the Canadian population,^47^ this study may have good generalizability to both the Canadian population and jurisdictions with similar universal single payer populations.

## 5 CONCLUSION

Although over time socioeconomic disparities in NSCLC diagnostic imaging, treatments and survival have not widened, inequalities among SES groups that have existed for over a decade, still persist today without improvement even within a universal health care setting. These disparities should remain a priority for lung cancer research and policymaking in future efforts to reduce inequalities in the NSCLC patient population. A better understanding of the underlying factors that affect imaging, treatment receipt, TTI, time to adjuvant treatment, and the causal pathway between SES and cancer survival is critical to both informing strategies aimed at narrowing the gaps between wealthier and poorer populations, and ensuring optimal outcomes and care for all patients.

## CONFLICT OF INTEREST

None declared.

## AUTHORS' CONTRIBUTIONS

Ms. Shah, Dr. Parmar and Dr. Chan have all made substantial contributions to the design of the work, interpretation of data, drafting, and critically revising the work.

## Supporting information

Table S1
