# Supplementary material for: Socioeconomic disparity trends in diagnostic imaging, treatments, and survival for non‐small cell lung cancer 2007‐2016
Source: Cancer Med. 2020 Mar 20;9(10):3407–16. doi: 10.1002/cam4.2978 (PMC7221447; doi:10.1002/cam4.2978)
Supplement: Supplementary file 1 — Table S1 [file CAM4-9-3407-s001.docx]

**Supplementary TABLE 1**. Administrative databases utilized for data collection

| Database | Description |
| --- | --- |
| Registered Persons Database (RPDB) | Includes information on patient demographics, geographic location, which allow for classification of socioeconomic status based on income of neighbourhood by postal code. |
| Ontario Cancer Registry (OCR) | Includes cancer best stage, date of last contact in hospital or death date.  Codes:  Topography – C340-C349  Morphology – 8010, 8012, 8020, 8040, 8042, 8046, 8070–8072, 8140, 8240, 8246, 8250, 8255, 8260, 8480, 8481, 8550, 8560, 8013, 8050, 8051, 8052, 8073, 8074, 8075, 8076, 8077, 8078, 8141, 8143, 8147, 8251, 8252, 8253, 8254, 8310, 8430, 8490, 8570, 8571, 8572, 8573, 8574, 8575 |
| New Drug Funding Program (NDFP) | Includes claims for anti-cancer therapies appearing funded through Cancer Care Ontario. Contains medication and clinical details. Used for the ascertainment of baseline characteristics (such as location of treatment etc.), the use of NDFP adjuvant chemotherapy drugs. |
| Ontario Health Insurance Plan (OHIP) Schedule of Benefits and Fees | Includes claims derived from anyone who can claim under OHIP (physicians, labs, external providers). Information includes service provided to patients, diagnoses, physician specialty, and date.  Codes:  Radiation – X310, X311, X312, X313  CT head – X188, X400, X401  MRI head – E875, E876, X421, X425  PET – J706  Bone scan – J650, J850, Y650, Y850, J651, J851, Y651, Y851 |
| Ontario Drug Benefit Claims (ODB) | Used for the ascertainment of drug claims and dates of services. Used dates of services.*  Drug Identification Number (DIN): EGFR-I – 2468050, 2248676, 2461862, 2461889, 2461870, 2454386, 2454394, 2269007, 2269023, 2269015, 2377713, 2377705, 2377691 |
| Canadian Institute for Health Information Discharge Abstract Database (CIHI-DAD) | Includes information on dates of patient hospital admission, diagnosis codes, lengths of stay, deaths in hospital and procedure codes. Used to ascertain hospitalizations and admission and discharge diagnoses.  Codes:  Surgery – 1GR87QB, 1GR87DA, 1GR87NW, 1GR89QB, 1GR91QB, 1GR89DA, 1GR89NW, 1GT87QB, 1GR91NWXXF, 1GT87NW, 1GT87DA, 1GT89QB, 1GT91QB, 1GT89NW, 1GT89DA, 1GT91NW |
| National Ambulatory Care Reporting System (NACRS) | Includes demographic, administrative, clinical and service-specific data for emergency department, day surgery and other ambulatory care visits. Used dates of visits.*  Codes:  Thoracic radiation – 1GT27JA, 1GT27JADA, 1GT27JADB, 1GT27JADC, 1GT27JADE, 1GT27JADG, 1GT27JX, 1GT27JXDC, 1GT27JXDE, 1GT27JXDG |

*Utilized OCR, NDFP, OHIP, ODB, CIHI-DAD, and NACRS to establish patient’s date of last contact
